# Early Detection of Response to Experimental Chemotherapeutic Top216 with [^18^F]FLT and [^18^F]FDG PET in Human Ovary Cancer Xenografts in Mice

**DOI:** 10.1371/journal.pone.0012965

**Published:** 2010-09-24

**Authors:** Mette Munk Jensen, Kamille Dumong Erichsen, Fredrik Björkling, Jacob Madsen, Peter Buhl Jensen, Liselotte Højgaard, Maxwell Sehested, Andreas Kjær

**Affiliations:** 1 Cluster for Molecular Imaging, Faculty of Health Sciences, University of Copenhagen, Copenhagen, Denmark; 2 Department of Clinical Physiology, Nuclear Medicine & PET, Rigshospitalet, Copenhagen, Denmark; 3 Topotarget A/S, Copenhagen, Denmark; Genentech, United States of America

## Abstract

**Background:**

3′-deoxy-3′-[^18^F]fluorothymidine (^18^F-FLT) is a tracer used to assess cell proliferation *in vivo*. The aim of the study was to use ^18^F-FLT positron emission tomography (PET) to study treatment responses to a new anti-cancer compound. To do so, we studied early anti-proliferative effects of the experimental chemotherapy Top216 non-invasively by PET.

**Methodology/Principal Findings:**

*In vivo* uptake of ^18^F-FLT in human ovary cancer xenografts in mice (A2780) was studied at various time points after Top216 treatment (50 mg/kg i.v. at 0 and 48 hours) was initiated. Baseline ^18^F-FLT scans were made before either Top216 (n = 7–10) or vehicle (n = 5–7) was injected and repeated after 2 and 6 hours and 1 and 5 days of treatment. A parallel study was made with 2′-deoxy-2′-[^18^F]fluoro-D-glucose (^18^F-FDG) (n = 8). Tracer uptake was quantified using small animal PET/CT. Imaging results were validated by tumor volume changes and gene-expression of Ki67 and TK1. Top216 (50 mg/kg 0 and 48 hours) inhibited the growth of the A2780 tumor compared to the control group (P<0.001). ^18^F-FLT uptake decreased significantly at 2 hours (−52%; P<0.001), 6 hours (−49%; P = 0.002) and Day 1 (−47%; P<0.001) after Top216 treatment. At Day 5 ^18^F-FLT uptake was comparable to uptake in the control group. Uptake of ^18^F-FLT was unchanged in the control group during the experiment. In the treatment group, uptake of ^18^F-FDG was significantly decreased at 6 hours (−21%; P = 0.003), Day 1 (−29%; P<0.001) and Day 5 (−19%; P = 0.05) compared to baseline.

**Conclusions/Significance:**

One injection with Top216 initiated a fast and significant decrease in cell-proliferation assessable by ^18^F-FLT after 2 hours. The early reductions in tumor cell proliferation preceded changes in tumor size. Our data indicate that ^18^F-FLT PET is promising for the early non-invasive assessment of chemotherapy effects in both drug development and for tailoring therapy in patients.

## Introduction

For the evaluation of effect in animal studies during preclinical development of new anticancer agents, reduction in tumor volume is the most commonly used criterion for efficacy. However, the time until tumor shrinkage can be long and it requires repeated tumor volume measurements several times weekly to show effect. Non-invasive molecular imaging such as positron emission tomography (PET) allows for biological processes to be visualized and quantified non-invasively over time. A non-invasive method to detect early biological response following anticancer treatment would be valuable in anticancer drug development to distinguish effective from non-effective drugs before changes in tumor volume become evident.

Increased cell proliferation is one of the main features of cancer [1]. Much research focuses on the non-invasive visualization of cell proliferation, which could be used to define a biological response to treatment early during the course of therapy. 3′-deoxy-3′-[^18^F]fluorothymidine (^18^F-FLT) is used as a PET tracer for visualization of cell proliferation [2]. ^18^F-FLT is a thymidine analogue and consequently a substrate of the DNA synthetic pathway [3]. When taken up in cells ^18^F-FLT is phosphorylated by thymidine kinase-1 (TK1), which leads to intracellular trapping. TK1 activity is tightly cell cycle regulated and is mainly expressed during the S-phase of the cell cycle; consequently, it is assumed to reflect the amount of proliferating cells [4], [5]. ^18^F-FLT uptake is positively correlated with cell growth and TK1 activity [5]–[7]. Several studies have showed a correlation between ^18^F-FLT uptake and tumor cell proliferation both in cancer xenografts in mice [8]–[11] and human tumor samples [12]–[14].

Several pre-clinical studies have evaluated proliferation measured by ^18^F-FLT PET in response to different chemo- and radiation therapies in different animal models of cancer [8]–[11], [15]–[19]. The results from these studies vary; the first change in ^18^F-FLT uptake is found in the range of 24 hours to one week after initiation of treatment. However, not all studies have found a correlation between tumor response and a change in ^18^F-FLT uptake after start of treatment [20], [21]. Seemingly, the time frame for assessing changes in proliferation is very variable according to different treatment regimes.

Early non-invasive detection of anti-proliferative activity with ^18^F-FLT PET could also be useful in a clinical setting to determine whether patients are responsive to conventional treatment and during phase I, II and III studies when evaluating responses to new anti-cancer drugs. Today the most widely used methods to assess tumor responses clinically is with anatomical imaging techniques such as computed tomography (CT) and magnetic resonance imaging (MRI) using the Response Evaluation Criteria In Solid Tumors (RECIST). However, this often requires several weeks or months before a possible response becomes evident [22], [23]. Recently, guidelines for analyzing tumor responses using PET and 2′-deoxy-2′-[^18^F]fluoro-D-glucose (^18^F-FDG) have been suggested stating the need for additional methods for measuring tumor responses [24]. New anti-cancer agents are often tumorstatic rather than tumoricidal and changes in tumor size can be minimal despite effective treatment thus generating a need for novel methods for assessing clinical response besides tumor volume shrinkage.

^18^F-FDG is currently the most widely used radiotracer for imaging in oncology and is very useful for detecting and characterizing cancers. Several studies have analyzed changes in ^18^F-FDG uptake following anti-cancer treatment, but with variable results [25]. ^18^F-FDG suffers from the limitation that it may not detect effects at an very early stage and an inflammatory response after cancer treatment may to some degree obscure the detection of anti-cancer effect [26], [27].

Top216 is a more potent and metabolically stable derivative of Top001, which was discovered by BioImage to have potent and selective killing effect on breast cancer cell lines [28]. Top001 inhibits the mTOR pathway in a cell-selective manner after prolonged incubation (∼24 hours). The correlation between mTOR inhibition and cell line sensitivity is good but not perfect.

Top216 inhibits protein, RNA, and DNA synthesis in sensitive cell lines after 1–2 hours of incubation and induces apoptosis. Induction of apoptosis as measured by caspase 3/7 activity is detectable after 6 hours in most sensitive cell lines. Top001 and Top216 do not significantly inhibit kinases (Upstate kinase panel) or receptors (Cerep panel) at relevant concentrations and at present the exact target or mode of action remains to be identified. Top216 shows potent *in vivo* efficacy in mouse xenograft models of human breast, prostate, ovarian, and pancreatic cancer, both when administered i.v. and p.o.. Top216 is currently undergoing regulatory safety and toxicology examination with the aim to move the compound into the clinic.

The aim of the study was to use ^18^F-FLT PET to study treatment responses to a new anti-cancer compound non-invasively. To do so we imaged cell proliferation *in vivo* with ^18^F-FLT PET in a human cancer mouse tumor model following initiation of treatment with Top216. Uptake of ^18^F-FLT was compared with uptake of ^18^F-FDG and Ki67 and TK1 gene expression.

## Materials and Methods

### Tumor model

Animal care and all experimental procedures were performed under the approval of the Danish Animal Welfare Council (2006/561-1124). Female NMRI (Naval Medical Research Institute) nude mice (8–11 weeks old) were acquired from Taconic Europe (Lille Skensved, Denmark) and allowed to acclimatize for one week in the animal facility before any intervention was initiated. The human ovarian carcinoma cell line A2780 (a gift from R. Ozols, Fox Chase Cancer Center Philadelphia, PA, January 2004) was used. 10^7^ cells in 100 µL medium mixed with 100 µL Matrixgel™ Basement Membrane Matrix (BD Biosciences, San Jose, CA, USA) were injected subcutaneously into the left and right flank respectively during anesthesia with 1∶1 v/v mixture of Hypnorm® (Janssen Pharmaceutica, Beerse, Belgium) and Dormicum® (Roche, Basel, Switzerland). The cell line has been tested free of mycoplasma; however, it has not been authenticated. Cells were cultured in RPMI (Roswell Park Memorial Institute) medium 1640+ GlutaMAX (Invitrogen, Carlsbad, CA, USA) supplemented with 10% fetal calf serum (Biological Industries, Israel) and 1% penicillin-streptomycin (Invitrogen) in 5% CO_2_ at 37°C.

### Experimental design

**Figure 1:**
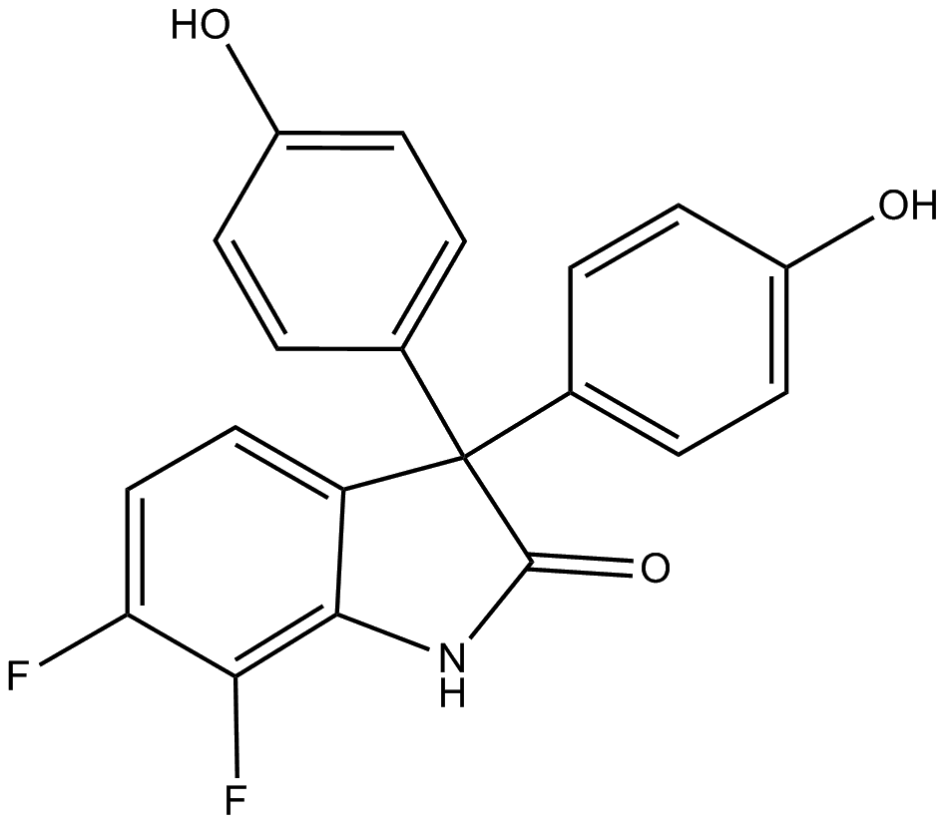
Chemical structure of Top216.

**Figure 2:**
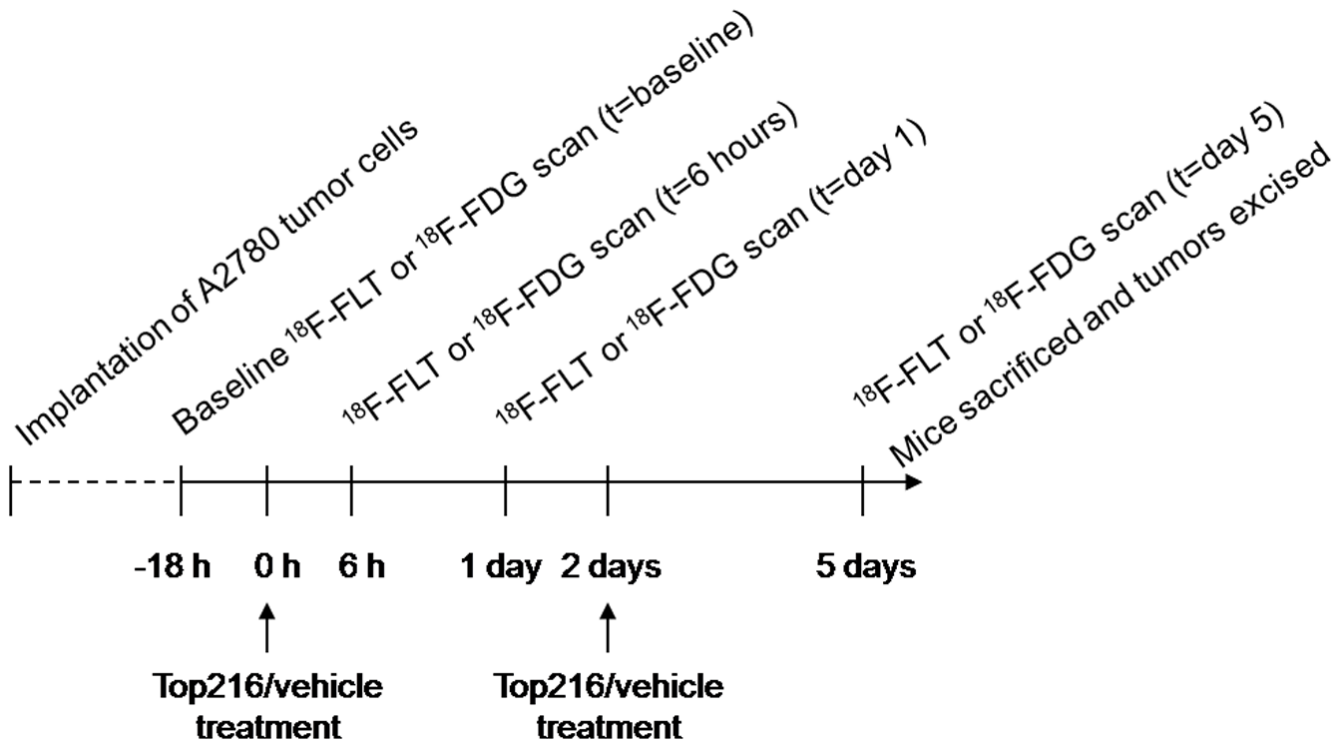
Schematic view of the experimental design.

Six groups of mice were followed (n = 5–10 tumors per group). Treatment was started at day 12–24 after implantation of tumor cells, when tumor volumes were on average 225 mm^3^. Mice received Top216 treatment 50 mg/kg i.v. or vehicle (2% DMSO, 20% HP-b-CD in saline) at 0 and 48 hours (figure 1). This dose of Top216 was in preceding analyses shown to inhibit growth of the A2780 xenograft tumor (data not shown). Before treatment was started, the mice were scanned with ^18^F-FLT or ^18^F-FDG in order to determine the baseline level of tracer uptake. ^18^F-FLT or ^18^F-FDG scans were repeated at 6, 30 (Day 1) and 126 (Day 5) hours after injection of Top216 or vehicle (figure 2) In addition, a group of mice was scanned with ^18^F-FLT at baseline and 2 hours after initiation of treatment (Top216 or vehicle). Tumor volume was followed by CT during the experiments [29]. Tumor volumes were calculated relative to volume at baseline.

Expression of Ki67 and TK1 was analyzed *in vitro* in a parallel group of mice, which were treated with either Top216 or vehicle and biopsied before and at 6, 30 (Day 1) and 126 (Day 5) hours after initiation of treatment. Biopsies were removed with an 18G needle and placed immediately in RNA later® (Ambion (Europe) Limited, Cambridgeshire, UK). All samples were stored at 4°C and the following day RNAlater® was removed and samples transferred to -80°C until further qPCR processing.

### Synthesis of ^18^F-FLT and ^18^F-FDG

[18F]FLT was synthesized using 3-N-Boc-1-[5-O-(4,4′-dimethoxytrityl)-3-O-nosyl-2-deoxy-b-D-lyxofuranosyl]thymine as precursor and synthesized on a GE TracerLab MX Synthesizer. All reagents and FLT cassettes were purchased from ABX (Radeberg, Germany). The radiochemical purity was determined after measuring the content of fluoride-18 and other radioactive impurities in the FLT solution measured with TLC and HPLC respectively. The content of ethanol and acetonitrile was determined by GC analysis. The pH was measured with a pH-meter. In separate preparations the stability of the preparations was examined after 8 hours. HPLC was performed on a Gilson HPLC system (Biolab A/S, Denmark) equipped with a Dionex UV-detector (Dionex Denmark A/S, Denmark) and an in-line radioactivity detector. The HPLC column was a Luna 5 µ C18(2) 100A, 150×4.6 mm (Phenomenex, Denmark). The eluent was water/acetonitrile 90/10 and a flow rate of 1 ml/min. UV detection at 267 nm. TLC plates were obtained from Merck and water/acetonitrile 5/95 was used as eluent. Residual solvents were determined on a Shimatzu GC 2014 (Holm & Halby, A/S, Denmark) equipped with a Chromosorb 101, 100–120 Mesh, 1/8″×10′ column, FID detector and helium carrier gas. The temperature of the column was 210°C. The radiochemical purity of ^18^F-FLT was >98% with a specific radioactivity ranging from 150–270 GBq/µmol at EOS. The ethanol content was in the range 7–8% and the amount of acetonitrile was below the detection limit. The pH was 7.5–7.8. The radiochemical purity, ethanol content and pH did not change after 8 hours of storage at room temperature.

^18^F-FDG was acquired from daily productions for clinical use (Rigshospitalet, Copenhagen, Denmark).

### microPET and microCT imaging

Mice were injected i.v. with 10.0±1.5 (mean ± SD) MBq ^18^F-FDG or 6.9±2.4 (mean ± SD) MBq ^18^F-FLT. Mice were fasted overnight before each ^18^F-FDG scan [30]. One hour after tracer injection mice were anaesthetized with 3% sevofluran (Abbott Scandinavia AB, Solna, Sweden) mixed with 35% O_2_ in N_2_ and fixed on a bed in presence of three fiducial markers allowing fusion of PET and CT pictures. A 20 min PET scan was acquired using a MicroPET Focus 120 (Siemens Medical Solutions, Malvern, PA, USA). After data acquisition, PET data were arranged into sinograms and subsequently reconstructed with the maximum a posteriori (MAP) reconstruction algorithm. The pixel size was 0.866×0.866×0.796 mm and in the center field of view the resolution was 1.4 mm full-width-at-half-maximum.

Following the microPET scan, a microCT scan was acquired with a MicroCAT® II system (Siemens Medical Solutions). A 7 minute and 10 seconds CT scan was performed with parameter settings: 360 rotation steps, tube voltage 60 kV, tube current 500 µA, binning 4 and exposure time 310 ms. The pixel size was 0.091×0.091×0.091 mm.

PET and microCT images were fused in the Inveon software (Siemens Medical Solutions). Before fusion region of interests (ROIs) were drawn on the CT pictures manually by qualitative assessment covering the whole tumors and subsequently tumor volume and tracer uptake, assessed by standard uptake values (SUV) mean and maximum, were generated by summation of voxels within the tomographic planes.

### Quantitative real-time polymerase chain reaction (qPCR)

Total RNA was isolated from the biopsies with TRI reagent® following the manufacturer's instructions (Molecular Research Center Inc., OH, USA) and subsequently RNA integrity was measured on a 2100 Bioanalyzer (Agilent Technologies, Santa Clara, CA, USA). RNA quality is stated as RNA integrity number (RIN) [31]. The concentration of the RNA was determined by NanoDrop 1000 (Thermo Fisher Scientific, Wilmington, DE, USA). Total RNA (0.3 µg) was reversed transcribed using the Affinityscript™ QPCR cDNA Synthesis kit (Stratagene, La Jolla, CA, USA) according to the manufacturer's instructions. Samples were cooled down and stored at −20°C until further use.

Gene expression was quantified on the Mx3000P® real-time PCR system from Stratagene. Ki67 and TK1 were each quantified in a duplex with TATA box binding protein (TBP). The Brilliant® QPCR Core Reagent Kit (Stratagene) was used. Optimization of assays resulted in 50% increase in dNTP and Taq polymerase. An MgCl concentration of 5.5 mM was used for all experiments. The following thermal profile was used in all experiments: 10 minutes of denaturation at 95°C followed by 45 cycles with denaturation for 30 seconds at 95°C and annealing/elongation at 60°C for 1 minutes.

Relative quantification by the comparative method (2^−ΔΔCt^) [32] was used. The measurements were corrected for the efficiency of the PCR reaction calculated by 5-fold dilution curves thereby replacing 2 in the formula with (E+1) [33]. Efficiency corrections were made for each gene in every assay. Tissue from baseline samples served as calibrator. TBP was used as reference gene. This gene was previously tested to be stable in tumor versus normal tissue and subsequently found to be stable in this experimental setup.

Primers and TaqMan dual-labeled probes were designed using Beacon Designer (PREMIER Biosoft, Palo Alto, CA USA). Primers and probes are shown in (table 1). For each gene the optimal primer and probe concentration was found. All samples were run in triplicate using one µl of cDNA. To each sample a no-reverse transcription control (NoRT) was included, and on each plate a no-template control (NTC) was included.

**Table 1:** Primer and probe sequences for the genes investigated.

Gene	Forward primer (5′-3′)	Reverse primer (5′-3′)	5′ fluorophore	Probe (5′-3′)	3′ Quencher	Amplicon length (bp)
TBP	tgttgagttgcagggtgtgg	tagcagcacggtatgagcaac	HEX	tgcccttctgtaagtgcccaccgc	BHQ-1	133
Ki67	tcccgcctgttttctttctgac	ctctccaaggatgatgatgctttac	FAM	agcccgatgccacccagtacagga	BHQ-1	121
TK1	gccgatgttctcaggaaaaagc	gcgagtgtctttggcatacttg	FAM	cgtccgtcgcttccagattgctca	BHQ-1	103

### Statistical analysis

Comparison of tumor volume between Top216 treated and control groups were calculated using a unpaired student's t-test. Paired t-test was used for intra-group comparisons. Bonferroni correction of p-values for multiple comparisons was applied. All data were tested to be normal distributed by means of Kolmogorov-Smirnov test. Calculations were made in SPSS 16.0. Data are reported as mean ± SEM and P<0.05 was considered statistically significant.

## Results

### Effect of Top216 on tumor size

**Figure 3:**
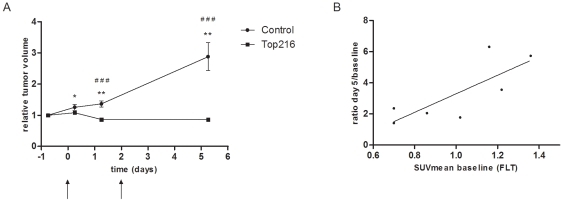
Tumor growth. A) The effects of Top216 on the growth of A2780 tumor xenografts. Tumor volume was determined by microCT. Mice were treated with Top216 (50 mg/kg) or vehicle at 0 and 48 hours. *) P<0.05, **) P<0.01 vs. baseline and ^# # #^) P<0.001 vs. control. n = 15 tumors per group. B) Changes in tumor volume assessed by ratio Day 5/baseline in the control group as a function of baseline FLT uptake. n = 7 tumors. R^2^ = 0.61, P = 0.04.

Top216 (50 mg/kg at 0 and 48 hours) inhibited the growth of A2780 human ovary cancer xenografts in mice *in vivo* compared with the control group (P<0.001) (figure 3). The sizes of the untreated tumors increased by approximately a factor 3 during the study and volumes of the treated tumors were unchanged.

### ^18^F-FLT and ^18^F-FDG uptake

Baseline tumor uptake of ^18^F-FLT in the A2780 tumor model was relatively high (SUVmean 1.05±0.03), making it easy to differentiate tumor from non-tumor tissues whereas for ^18^F-FDG only a modest tumor uptake was observed (SUVmean 0.48±0.02). In the control group, baseline ^18^F-FLT uptake predicted tumor volume increase over 5 days (linear regression of SUVmean baseline vs. tumor volume ratio Day 5/baseline: r^2^ = 0.61, P = 0.04) (figure 3). No correlation between baseline ^18^F-FDG uptake and tumor volume increase was observed.

**Figure 4:**
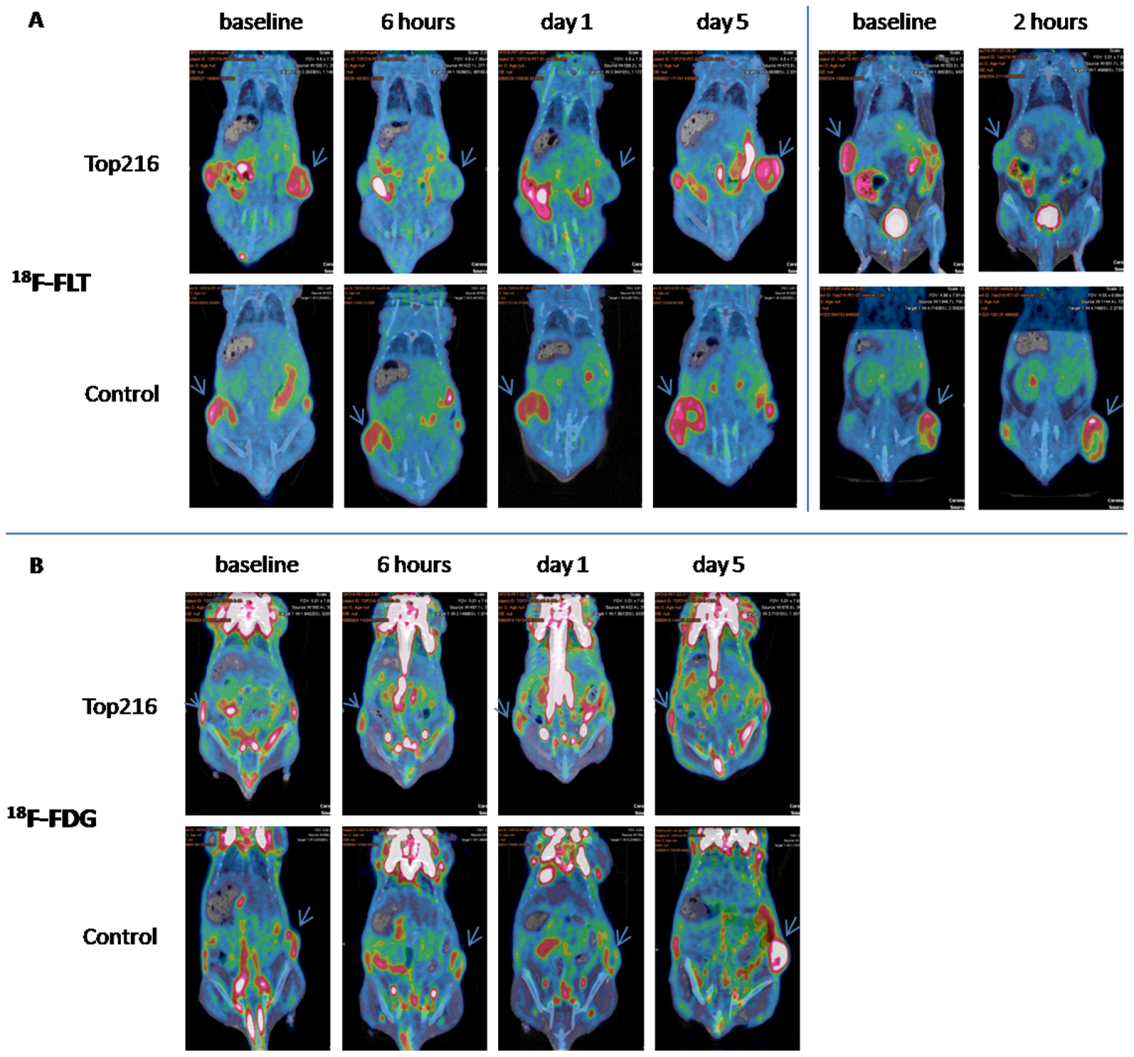
Fused PET/CT images. A) The eight images at left are representative coronal fused PET/CT images of two mice scanned with ^18^F-FLT at baseline and at 6 hours and 1 and 5 days after treatment start. The images at the top show one mouse treated with Top216 and the images at the bottom show one control mouse which received vehicle. The four images at right show fused PET/CT pictures of two representative mice treated with either Top216 or vehicle and scanned at baseline and 2 hours after treatment initiation. The arrows point towards the tumors. B) Representative coronal fused PET/CT images of two mice scanned with ^18^F-FDG at baseline and 6 hours and 1 and 5 days after treatment start. The images at the top show one mouse treated with Top216 and the images at the bottom show one control mouse which received vehicle. The arrows point towards the tumors.

Uptake of ^18^F-FLT assessed by SUVmean decreased significantly from 1.09±0.03 at baseline to 0.53±0.02 (−52%; P<0.001) at 2 hours, to 0.56±0.06 (−49%; P<0.001) at 6 hours and to 0.58±0.03 (−47%; P<0.001) at Day 1 after Top216 treatment initiation (figure 4+5).

**Figure 5:**
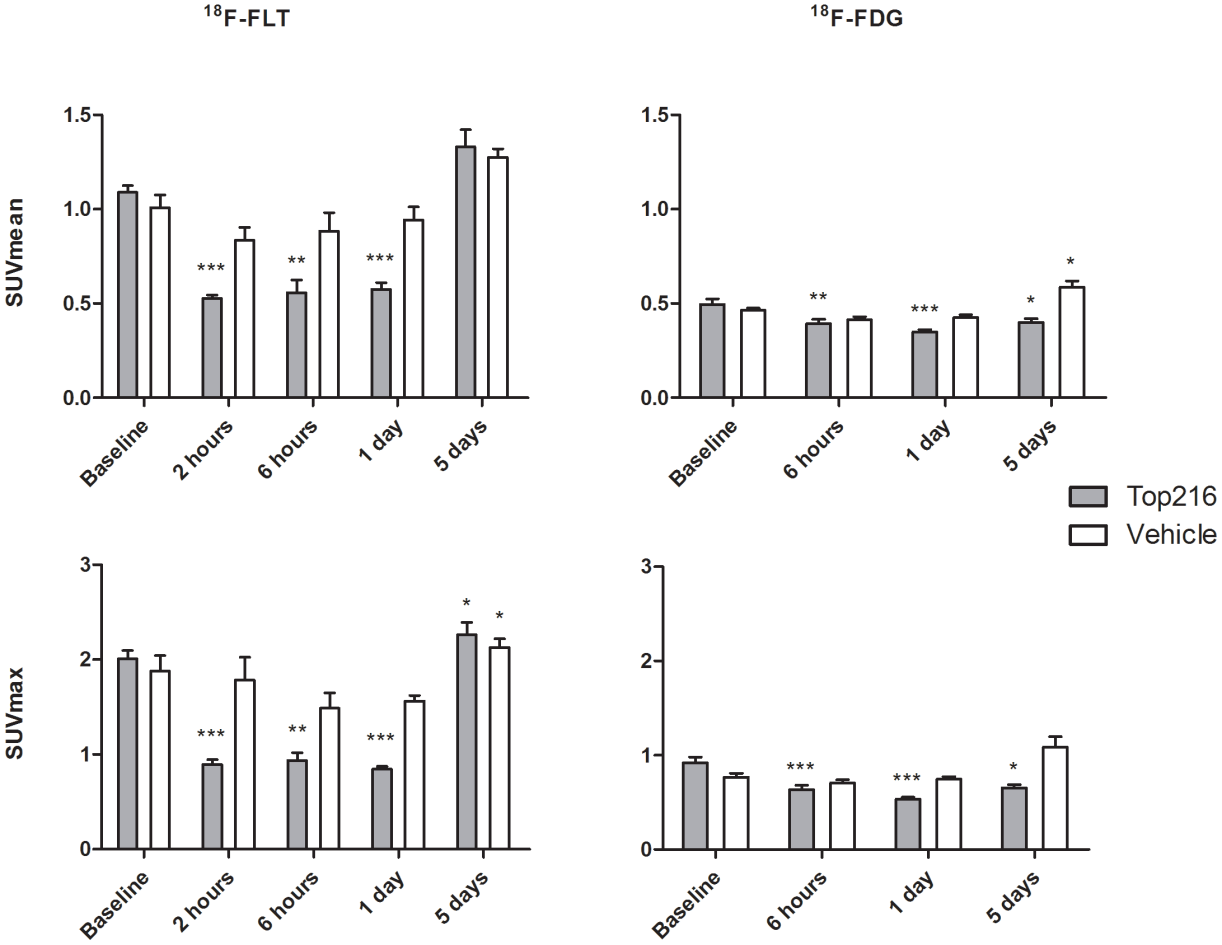
^18^F-FLT and ^18^F-FDG uptake assessed by SUVmean and SUVmax at baseline and following treatment with Top216. Top216/vehicle treatment was initiated at 0 hours and repeated at 48 hours after the first injection. N = 5–10 tumors per group. *) P<0.05, **) P<0.01, ***) P<0.001 compared to baseline. The two graphs at left show data from the ^18^F-FLT experiments and the two graphs at right show data from the ^18^F-FDG experiments.

After 5 days ^18^F-FLT uptake (1.33±0.08) was comparable to baseline uptake. SUVmean was unchanged in the control group during the experiment. SUVmax values decreased significantly from 2.01±0.09 at baseline to 0.89±0.05 at 2 hours (−55%; P<0.001), to 0.94±0.08 at 6 hours (−53%; P = 0.002) and to 0.84±0.03 at Day 1 (−58%; P<0.001). SUVmax values increased to 2.26±0.12 at Day 5 after treatment initiation (13%; P = 0.04). SUVmax was unchanged in the control group, however increased slightly at Day 5 after treatment initiation compared to baseline (13%; P = 0.03).

Uptake of ^18^F-FDG assessed by SUVmean decreased significant from 0.49±0.03 at baseline to 0.39±0.02 (−21%; P = 0.003) at 6 hours, to 0.35±0.01 (−29%; P<0.001) at Day 1 and to 0.40±0.02 (−19%; P = 0.05) at Day 5 (figure 4+5). Uptake of ^18^F-FDG in the control group was significantly increased at Day 5 compared to baseline uptake (27%; P = 0.05). SUVmax values decreased significantly from 0.92±0.06 at baseline to 0.64±0.04 at 6 hours post injection (−31%; P<0.001), to 0.53±0.02 at Day 1 (−42%; P<0.001) and to 0.66±0.03 at Day 5 (−29%; P = 0.01). SUVmax was unchanged in the control group during the experiment.

### Expression of Ki67 and TK1

Expression of the reference gene TBP was constant throughout the experiment. Differences in Ct-values between normal samples and NoRT samples were 13 (median). RNA integrity numbers (RIN-values) were 9.1±0.1 (mean±SD) for all samples.

**Figure 6:**
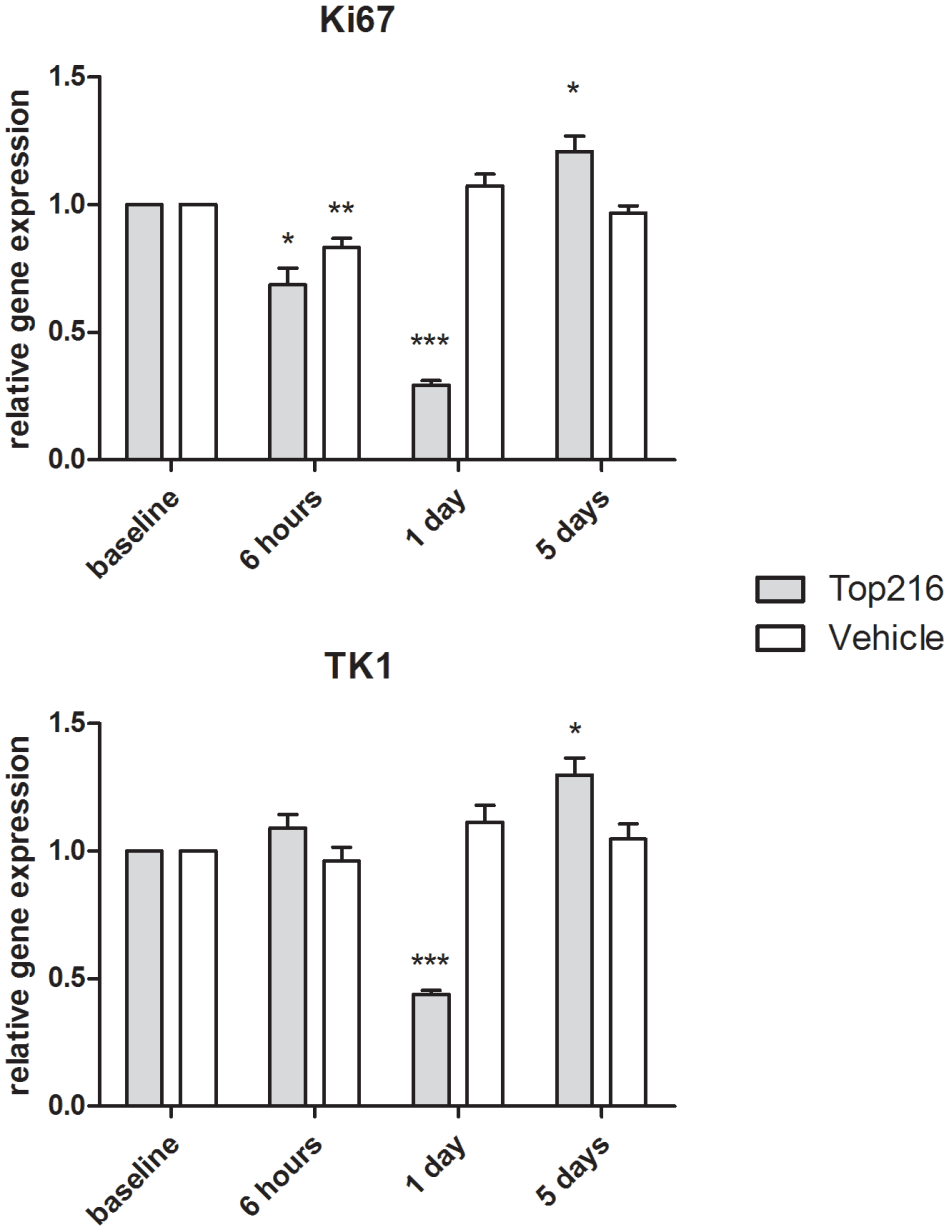
Expression of Ki67 and TK1 normalized to expression of reference gene TBP. Data are presented as fold changes following treatment with Top216/vehicle relative to baseline levels (n = 7 tumors per group). Top216 treatment was initiated at 0 hours and repeated at 48 hours after the first injection. *) P<0.05, **) P<0.01, ***) P<0.001 compared to baseline.

Gene expression levels of Ki67 and TK1 are shown in figure 6. In the treatment group expression of Ki67 was significantly lower at 6 hours post injection (−31%, P = 0.01) and Day 1 (−71%; P<0.001) after treatment start compared to baseline. Expression of Ki67 was 21% increased at Day 5 (P = 0.04) compared to baseline. Expression of Ki67 in the control group was significantly lower at 6 hours (−17%; P = 0.01) compared to baseline.

In the treatment group expression of TK1 was significantly decreased at Day 1 (−56%, P<0.001) compared to baseline. At Day 5 expression of TK1 was increased (30%, P = 0.013) compared to baseline. In the control group expression of TK1 was unchanged during the experiment.

## Discussion

One injection with Top216 initiated a fast and significant decrease in cell proliferation of 52% assessable by ^18^F-FLT PET as early as 2 hours post-injection. This decrease lasted for at least 1 day, but on Day 5 (3 days after the 2^nd^ treatment) uptake of ^18^F-FLT was comparable to uptake in the control group suggesting that the tumor cells had regained their proliferation capacity. Uptake of ^18^F-FLT in the control group did not change during the experiment thus validating the anti-proliferative effect of Top216. In contrast to the steep decrease in ^18^F-FLT uptake following treatment start, a small, but significant, decrease in ^18^F-FDG uptake was observed at 6 hours and on Day 1 after initiation of Top216 treatment, and this decrease lasted until Day 5. Changes in ^18^F-FLT uptake (max decrease: 52%; 2 h) were more pronounced than changes in ^18^F-FDG uptake (max decrease: 29%; Day 1). However, at the end of the experiment, ^18^F-FDG uptake remained lower than at baseline in the Top216 group, whereas it was increased in the control group.

The decrease in ^18^F-FLT uptake early post-injection was not accompanied by a decrease in tumor volume as volume of the tumors did not change during the course of therapy. This illustrates that the use of non-invasive imaging to assess tumor response is important since evaluating reduction in tumor volume as an endpoint would have generated a false negative conclusion. Anti-volume effect of Top216 was, however, still seen compared with the control group. Changes in tracer availability e.g. as a consequence of tumor perfusion alterations after treatment, could account for some of the effect on decreased ^18^F-FLT uptake. However, the fact that ^18^F-FDG uptake did not decrease the same way as ^18^F-FLT leads to the assumption that decrease in ^18^F-FLT uptake is not only due to a change in tumor perfusion but also to a physiologic change in tumor cell proliferation. This was further validated by a similar decrease in Ki67 gene expression.

The measurements of tracer uptake on Day 5 can be interpreted both as a measure of cell proliferation 5 days after treatment initiation and as proliferation status 3 days after the second injection of Top216. Consequently, one injection of Top216 inhibited proliferation somewhere between 1 and 3 days, thereafter the tumor cells recovered their proliferation capacity. This information could be useful when planning treatment schedules during pre-clinical investigations and in future clinical protocols in order to find the optimal treatment schedule.

On Day 5 after treatment initiation ^18^F-FDG uptake was 19% lower compared to baseline, whereas uptake of ^18^F-FLT was comparable to baseline. Uptake of ^18^F-FDG was consequently affected for a longer time than ^18^F-FLT uptake. This indicates that even though cell proliferation after 5 days (3 days after the 2^nd^ treatment) was comparable to baseline, biological effects of the treatment still existed and were visualized by ^18^F-FDG. Uptake of ^18^F-FDG was significantly lower at 6 hours after start of treatment compared to baseline uptake. However the decrease of 21% from an already low uptake is not easily visualized on the PET/CT images (figure 4).

Our findings that ^18^F-FLT was superior to ^18^F-FDG for assessing the early responses following anti-cancer treatment are in agreement with other studies [8], [11], [21]. Baseline levels of ^18^F-FDG uptake in the tumor model were low and only about half of the baseline levels of ^18^F-FLT uptake which is in agreement with the findings of others [11], [17]. However, other studies have found a higher ^18^F-FDG uptake in several xenograft models compared to ^18^F-FLT uptake [16], [19], [21]. It has previously been shown that it is more difficult to measure treatment response in tumors with low baseline tracer uptake, which is in concordance with the results found in this study [11], [21], [34].

The findings from other studies using ^18^F-FLT PET to assess early responses to anti-cancer treatment have been very variable, where response to a histone deacetylase inhibitor was observed after 4 days [10], response to cisplatin is seen after 1 day [9], response to cyclophosphamide and mTOR inhibition is evident after 2 days [16] and the ErbB kinase inhibitor initiated a decrease in ^18^F-FLT uptake 2 days after treatment initiation whereas no response was observed at 6 and 24 hours [11]. However comparison of the studies is difficult due to different treatment and scanning schedules and variable tumor models. Compared to other studies we found a steep decrease in ^18^F-FLT uptake assessed by PET and this decrease was observed much earlier (2 and 6 hours). It remains to be established whether this early response is compound specific or simply due to our protocol being the first to assess response so early after treatment.

Whether or not the early change in ^18^F-FLT and ^18^F-FDG can be a predictor of clinical outcome is still unknown and further studies investigating early changes and overall survival are needed in order to answer that question.

Comparison of ^18^F-FLT uptake and Ki67 gene expression showed a similar change following treatment with Top216. However, Ki67 mRNA levels did not decrease as much as ^18^F-FLT uptake 6 hours after treatment initiation. A possible explanation could be that changes in enzymatic activity occur before changes in mRNA levels. The correlation between Ki67 gene expression and ^18^F-FLT uptake in our study is in accordance with other studies finding strong correlation between Ki67 at the protein level and ^18^F-FLT uptake [8]–[11]. We found a significant decrease in ^18^F-FLT uptake as early as 2 and 6 hours after treatment initiation; however, despite a significantly lower ^18^F-FLT uptake at 6 hours, a decrease in TK1 gene expression was first evident on Day 1 after treatment initiation. TK1 enzyme activity is positively correlated with ^18^F-FLT uptake [6], [7] and it has been shown that transcriptional mechanisms may take part in regulation of TK1 activity where both TK1 protein and mRNA levels were related to a decrease in ^18^F-FLT uptake following treatment with a histone deacetylase inhibitor [10]. Early changes in ^18^F-FLT uptake without changes in TK1 mRNA levels are likely due to changes in protein levels, posttranslational protein modifications or changes in ATP levels [7], [10], [35]. ATP is required for TK1 activity [36] and other studies have similarly found a decreased ^18^F-FLT uptake, despite a high TK1 level, which could be explained by a low level of ATP [8].

In conclusion, we found a 52% decrease in ^18^F-FLT uptake as early as 2 hours after the first injection of Top216. ^18^F-FLT was superior to ^18^F-FDG as a noninvasive tool to assess early biological responses to Top216. Decrease in ^18^F-FLT uptake preceded reductions in tumor growth. The results from this study show the possibility of using non-invasive ^18^F-FLT PET to evaluate responses during development of new anti-cancer agents and of following treatments without the need to acquire serial tumor biopsies or waiting weeks or months before a possible tumor reduction is seen.
